# Statistical data for the tensile properties of natural fibre composites

**DOI:** 10.1016/j.dib.2017.03.043

**Published:** 2017-04-08

**Authors:** J.P. Torres, L.-J. Vandi, M. Veidt, M.T. Heiztmann

**Affiliations:** The University of Queensland, Australia

## Abstract

This article features a large statistical database on the tensile properties of natural fibre reinforced composite laminates. The data presented here corresponds to a comprehensive experimental testing program of several composite systems including: different material constituents (epoxy and vinyl ester resins; flax, jute and carbon fibres), different fibre configurations (short-fibre mats, unidirectional, and plain, twill and satin woven fabrics) and different fibre orientations (0°, 90°, and [0,90] angle plies). For each material, ~50 specimens were tested under uniaxial tensile loading. Here, we provide the complete set of stress–strain curves together with the statistical distributions of their calculated elastic modulus, strength and failure strain. The data is also provided as support material for the research article: “The mechanical properties of natural fibre composite laminates: A statistical study” [1].

**Specifications table**

**Table:** 

Subject area	Materials Science
More specific subject area	*Natural fibre composites*
Type of data	*Text file, image*
How data was acquired	*Uniaxial tensile testing performed on an INSTRON 5584 frame*
Data format	*Raw, analyzed*
Experimental factors	*Rectangular samples cut from vacuum infused laminates*
Experimental features	*Large statistical sets of several natural fibre reinforced composite laminate systems*
Data source location	http://dx.doi.org/10.17632/v25pzywt5c.1
Data accessibility	*Data are presented in this article*
Related research article	*“J.P. Torres, L.-J. Vandi, M. Veidt, M.T. Heitzmann. The mechanical properties of natural fibre composite laminates: A statistical study. Composites Part A: Applied Science and Manufacturing, Volume 98, July 2017, Pages 99-104, ISSN 1359-835X, http://doi.org/10.1016/j.compositesa.2017.03.010.*

**Value of the data**

• The statistical distributions presented here can be used to model the probability of failure of material properties in similar material systems.
• These mean and standard deviation values of mechanical properties can be used to determine design allowables and confidence levels in component design.
• This data is useful to compare the variability of mechanical properties of natural fibres to other types of reinforcement fibres when assembled in composite laminates.
• Allows calculating Weibull probability distribution parameters for a given distribution using a rank regression method.

## 1 Data

This article features raw stress–strain tensile data for approximately 500 specimens corresponding to different natural fibre reinforced composite laminates. In addition, we provide here the calculated elastic modulus, strength and failure strain values for each specimen. Finally, we include python codes that enables to show the experimental statistical distributions for each material system and calculate the corresponding fit of their probability distribution functions. The complete data can be found in the file ‘*Data_in_Brief-Natural_Fibres.zip’* available in the Mendeley data repository under the following identifier DOI: 10.17632/v25pzywt5c.1.

## 2 Experimental design, materials and methods

### 2.1 Materials

**Fig. 1:**
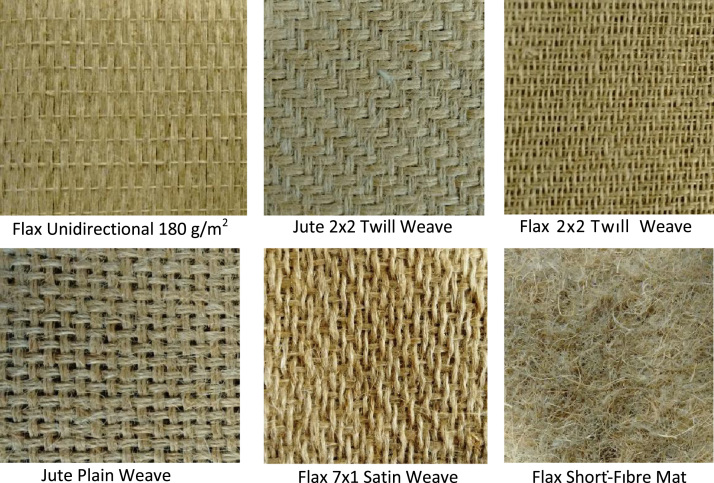
Natural fibre fabric configurations.

Several natural fibre reinforced composites laminates were tested under uniaxial tensile loading. Table 1 shows the specifications for each material including: fibre and matrix characteristics, fibre orientation, fabric configuration, laminate thickness and fibre volume fraction. Fig. 1 shows images of the fiber and fabric configurations. The laminates were manufactured using vacuum assisted resin infusion. Table 2 shows the specimen manufacturing process details.

### 2.2 Tensile testing

**Fig. 2:**
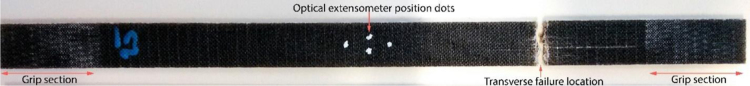
Tested Flax-0 specimen showing failure location.

We carried out uniaxial tensile tests were an electromechanical INSTRON 5584 frame using a 30 kN load cell following the procedures depicted in [9]. Tensile specimens were supported with hydraulic grips with a 17.5 MPa gripping pressure that prevented both specimen sliding and premature failure at the grips. Both longitudinal and transverse engineering strains were measured using an optical extensometer. Observed failure for all systems was brittle fracture in the gage section (Fig. 2).

The raw data presented in this article are Comma Separated Values (.csv) files generated by the Instron Bluehill testing software [8] containing columns for Test Time, Extension (i.e. crosshead displacement), Load, Axial and Transverse strains (measured with optical extensometer), and Stress (calculated by dividing the applied load by the initial cross section area). In addition, each file contains a header showing specimen thickness and width, axial and transverse initial gage length for the extensometer, and an automated Bluehill calculation of the elastic modulus and Poison׳s ratio (please note that these last two values were not used in the generation of material properties, instead values were recalculated from the raw stress–strain data). Each material system contains a file folder labeled following the convention presented in Table 1. These folders contain the.csv files corresponding to each specimen and labeled as *Specimen_RawData_i*, were subscript *i* indicates the specimen number. All the raw data folders can be found in the compressed Raw Data folder.

In addition, we include a *Statistics* folder that incorporates the results from the analyzed elastic modulus, strength and failure strain data for each material system. Finally, we also include a Python script file used for the calculation of the statistical parameters and probability distribution functions (see following section).

### 2.3 Data processing

For each stress–strain curve, elastic modulus, strength and failure strain were calculated as follows. Elastic modulus was determined from the initial slope of the experimental stress–strain curves in the strain range 0.001–0.003 mm/mm (with the exception of material Flax-0 which used a 0.002–0.004 range to account for a larger initial toe-region). Strength and strain at failure were determined at the point corresponding to the maximum axial load in the stress–strain curves. For the postprocessing of all results, a preload stress level of 5 MPa was used to cut irrelevant data corresponding to the initial specimen+grips alignment. This means that all the measurements that occurred before reaching an applied stress of 5 MPa were not considered and all values were reset to zero at this point.

The statistical analysis presented in the accompanying research article [1] involves the determination of arithmetic mean, standard deviation and coefficient of variation. Automated calculation of these values is readily available in a vast number of commercial software programs. Calculation of the 2-parameter Weibull probability distribution function (PDF) parameters was carried out using the Median Rank regression technique [10]. *Accompanying this article is the python code used to calculate Weibull PDF parameters, under the name ‘Weibull.py’*.

This technique implies calculating the Median Rank position (MR) for each strength or failure strain value as follows:

$$\mathrm{MR}(i)=\;\frac{i-0.3}{N+0.4}$$

where *i* is the order number (i.e. the ordinal position of the mechanical variable in the entire sample) and *N* is the sample size. Next, Weibull parameters are calculated using the linear regression method, which involves calculating the following *N*-sized vectors:

$$y_{i}=\ln\left(-\ln\left(1-\mathrm{MR}(i)\right)\right)$$

$$x_{i}=\ln f_{i}$$

where *f*_*i*_ is the vector containing the discrete strength or failure strain values. Vectors *x*_*i*_ and *y*_*i*_ allow calculating parameters $\hat{b}$ and $\hat{a}$ as follows:

$$\hat{b}=\frac{\sum_{i=1}^{N}x_{i}y_{i}-\frac{\sum_{i=1}^{N}x_{i}\sum_{i=1}^{N}y_{i}}{N}}{\sum_{i=1}^{N}x_{i}^{2}-\frac{{\left(\sum_{i=1}^{N}x_{i}\right)}^{2}}{N}}$$

$$\hat{a}=\frac{\sum_{i=1}^{N}y_{i}}{N}-\hat{b}\frac{\sum_{i=1}^{N}x_{i}}{N}$$

Finally, the Weibull distribution parameters, *η* and *β,* are directly determined from $\hat{b}$ and $\hat{a}$ following:

$$\beta =\hat{b}$$

and

$$\eta =e^{-\frac{\hat{a}}{\hat{b}}}$$

where *β* is the shape parameter (Weibull modulus) and *η* is the scaling parameter.

## Figures and Tables

**Table 1:** Material specifications.

**Fibers**			**Matrix**		**Number of plies**	**Fibre volume Fraction**	**Label**
**Material**	**Configuration**	**manufacturer**	**Material**	**manufacturer**			
Flax 180 g/m^2^	0° UD	Lineo [2]	Epoxy SR-8100	Sicomin [4]	3	0.30	Flax-0
	90° UD				4	0.31	Flax-90
	[0/90]_2s_				4		Flax-CP
Flax 400 g/m^2^	7×1 Satin Weave	Biotex [3]			4	0.35	Flax-Satin
Flax 200 g/m^2^	2×2 Twill Weave				4	0.35	Flax-Twill
Jute 550 g/m^2^	2×2 Twill Weave				4	0.36	Jute-Twill
Jute 500 g/m^2^	Plain Weave				4	0.40	Jute-Plain
Flax 180 g/m^2^	0° UD	Lineo [2]	Vinyl Ester KRF2000SE	EPOVIA [5]	3	0.32	Flax-VE-0
Flax Mat	Random short-fibre mat	CIC (Non-commercial) [6]	Epoxy SR-8100	Sicomin [4]	2	0.25	Flax-Short
Carbon Fibre 300 g/m^2^ (UT-C300)	0° UD	Gurit [7]			6	0.48	Carbon-0

**Table 2:** Specimen manufacturing specifications.

Laminate manufacturing method	Vacuum assisted resin infusion
Fibre drying (before infusion)	6 h at 60 °C
Laminate post-curing	8 h at 60 °C and 80 kPa vacuum
Specimen size	250 mm×25 mm rectangular
